# 971. Enhancing Post Operative Rehabilitation Outcomes After Burn Scar Releases: A Retrospective Analysis

**DOI:** 10.1093/jbcr/irag033.568

**Published:** 2026-04-06

**Authors:** Karen E Robertson, Caroline Patterson, Fatih Zor

**Affiliations:** Riley Hospital for Children at Indiana University Health, Indianapolis, IN; Riley Hospital for Children at Indiana University Health, Indianapolis, IN; Indiana University Health, Indianapolis, IN

## Abstract

**Introduction:**

This study evaluated factors influencing therapy needs and complexity of care following pediatric burn reconstruction, with emphasis on areas where collaboration and prehabilitation could reduce challenges.

**Methods:**

A review of the Burn OR schedule in the electronic medical record was conducted to identify burn reconstruction cases from July 2022 through December 2024. A retrospective review identified 73 patients who underwent 104 scar releases in this time frame. Data included burn size, release type/location, timing of procedures, therapy frequency and setting, interventions (pre and post surgery), range of motion, scar scores, and complications.

**Results:**

Patients with dermal substitutes were associated with unexpected initial range of motion loss in both surgical and adjacent areas due to pain and delayed autografting, as well as prolonged immobility, splinting, and underlying joint/tendon tightness. In all cases, releases ultimately improved cutaneous mobility but required extensive postoperative therapy to address tightness of deeper structures and maintain integumentary range. Suboptimal positioning, simultaneous anterior/posterior releases, and closely spaced procedures (especially in the hands) limited functional gains. Patients with extensive burns also struggled to prioritize rehabilitation across multiple surgical sites.

**Conclusions:**

Rehabilitation following scar release is hindered by adjacent joint restrictions, positioning limitations, and competing therapy demands. Anticipating these barriers through prehabilitation, comprehensive therapy perspective, and close surgical–therapy collaboration may ultimately reduce therapy frequency and improve outcomes.

Limitations and opportunities for future research:

This single-center retrospective study was limited by a modest sample and assessment primarily through range of motion and scar scales rather than standardized functional measures. Future research should include prospective, multi-center studies with validated outcomes to quantify recovery and recurrence of contracture and assess the impact of prehabilitation, optimized positioning, and coordinated surgical–therapy planning.

**Applicability of Research to Practice:**

-Provide structured prehabilitation to optimize mobility and prepare for postoperative demands.

-Tailor positioning to stretch tendons, muscles, and cutaneous units while protecting uninvolved areas.

-Coordinate surgery timing to avoid procedures that hinder rehabilitation.

-Involve therapists early in postoperative care to guide scar management and continuity with community providers.

**Funding for the study:**

N/A.

